# Supplementary material for: Deformation- and damage-free transfer of soft electronics onto highly curved and fragile biological surfaces
Source: Nat Commun. 2026 Mar 26;17:4448. doi: 10.1038/s41467-026-70948-5 (PMC13183984; doi:10.1038/s41467-026-70948-5)
Supplement: Supplementary file 1 — Supplementary Information [file 41467_2026_70948_MOESM1_ESM.pdf]

Supplementary Materials for

Deformation- and Damage-Free Transfer of Soft Electronics onto Highly Curved and Fragile Biological Surfaces

Kyeong Min Song[1,2,#], Myung-Kun Chung[3,#], Jeehoon Jung[4], Jungjae Park[5], Min-Uk Kim[3], Jae-Young Yoo[6], Minsu Park[7], Geumbee Lee[8], Jeehyun Hong[2], Jun-Bo Yoon[3,*], and Yeon Sik Jung [2,*]

#These authors contribute equally to this work.
[*]Email: jbyoon@kaist.ac.kr (J.-B.Y), ysjung@kaist.ac.kr (Y.S.J)

The PDF file includes:

Supplementary Notes 1 to 7
Supplementary Tables 1 to 5
Supplementary Figs. 1 to 17
Supplementary References 1 to 36

Other Supplementary Materials for this manuscript include the following:

Supplementary Movies 1 to 2

Supplementary Note 1 | Strategies for conformal integration on curved biological surfaces

1 Existing approaches for integrating electronics onto curved biological surfaces (Supplementary Table 1)

A wide range of strategies has been developed to integrate flexible or stretchable electronic devices onto curved and dynamically contoured biological surfaces. Representative approaches include: (1) Halographic lithography, (2) Direct 3D printing (inkjet, aerosol jet, spray), (3) Deformable substrates (stretchable and thermoforming), (4) Shape reconfiguration. Although these strategies demonstrate varying degrees of conformal coverage, none can fully preserve the structural sophistication and alignment fidelity of microfabricated electronics required for high performance electronic systems on challenging biological geometries.

2 Transfer printing as the only route compatible with semiconductor microfabrication (Supplementary Table 1)

In contrast to the above approaches, transfer printing provides the only broadly applicable route for integrating semiconductor microfabricated devices onto complex three dimensional biological surfaces. Its key advantage arises from decoupling high precision device fabrication from the integration process. Semiconductor devices can first be fabricated on planar wafers using established lithographic processes, ensuring exceptional electrical performance, submicron resolution, and multilayer architectural control. These devices are then detached from the wafer and deterministically transferred onto soft or curved biological targets using an intermediate carrier. Because transfer printing preserves the original microfabrication fidelity while allowing placement onto nonplanar surfaces, it achieves levels of complexity and performance that are unattainable with direct fabrication or printing based methods. As a result, transfer printing uniquely enables reliable integration of ultrathin semiconductor devices with strongly curved, fragile, or anatomically challenging biological surfaces.

Supplementary Table 1 | Comparison of representative strategies for conformal integration of electronic devices on curved biological surfaces. Five representative fabrication routes for integrating electronic devices onto curved biological surfaces are summarized. Each strategy is compared in terms of its working principle, compatibility with semiconductor devices.

Strategy	Schematics	Operating principle	Compatibility with semiconductor devices	Ref.
			**Accurate biological sensing** and **multifunctional integration.**	
**Halographic lithography**	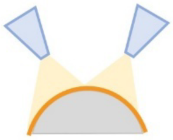	Uses laser interference patterns to define 3D or periodic microstructures on curved photoresist surfaces	**Low** Limited for multilayer semiconductor stacks or integrated circuits	1
**Direct 3D printing (Inkjet, aerosol jet, spray)**	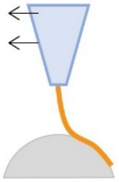	Deposits conductive or functional inks directly onto curved substrates	**Low** Difficult to achieve multilayer or high-density integration	2-4
**Deformable substrate (Stretchable, thermoforming)**	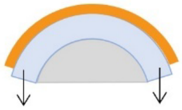	Employs substrates that stretch or thermally deform to match surface curvature	**Moderate** Limited by mechanical strain and long-term reliability	5-8
**Shape reconfiguration**	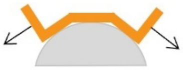	Transforms planar devices into 3D geometries via mechanical relaxation, folding, or thermal shrinking	**Moderate** Geometry-dependent and less suitable for fragile biological surfaces	9-12
**Transfer printing**	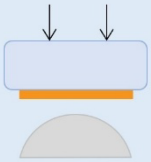	Pre-fabricated devices are relocated via temporary carriers.	**High** Only universal route for integrating wafer-grade microdevices onto 3D curved biological substrates	13-20

Supplementary Table 2 | Comparative analysis of existing transfer printing carriers and the unmet need for an adaptive medium. This table summarizes representative transfer printing carriers, their contact-to-removal operating principles, and their applicability in terms of maintaining the fidelity of soft electronics on curved surfaces and ensuring the safety of fragile biological tissues.

Transfer printing carrier	Operating principle (Schematic: contact → removal)	Applicability		Ref.
		Fidelity of soft electronics on high curvature	Safety of fragile biological surface	
**Elastomeric stamp (e.g., PDMS)**	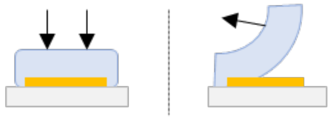 Pick-up and release driven by pressure-induced kinetic adhesion and contact	**Low** Tensile strain causing misalignment of ultrathin devices.	**Low** Pressure stress inducing surface deformation	13-15
**Adhesive-film**	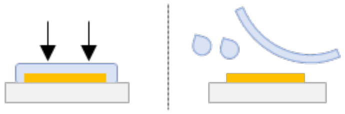 Temporary adhesion switch using water -sensitive or UV-releasable films	**Low** Limited coverage of highly curved surfaces	**High** Gentle contact and clean removal	16
**Thermal phase-change carrier (e.g., molten sugar)**	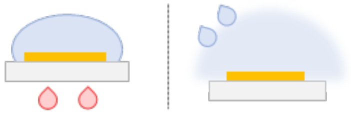 Solid–liquid transition triggered by heating and cooling cycles	**High** Conformal adaptation in molten state	**Low** Thermal damage	17-19
**Hydrotransfer carrier**	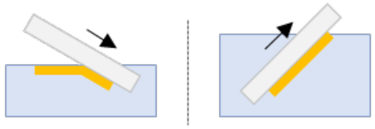 Surface tension and capillary flow driving adhesion and release	**Low** Uncontrolled fluid motion causing pattern drift	**High** Gentle contact mi nimizing mechanic al stress	20
**DAYS-fluid (this work)**	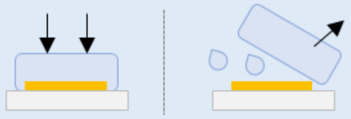 Stress-responsive rheology with reversible solid–liquid transition and water-triggered adhesion switching	**High** Conformal, strain-free contact on complex curvatures	**High** Non-invasive transfer	

Supplementary Table 3 | Key mechanical implications of DAYS-fluid versus sugar-transfer for microdevice integration on highly curved biological surfaces[1-5]**.** Comparison of DAYS-fluid and sugar-transfer in terms of their mechanical suitability for transferring microfabricated devices onto highly curved and fragile biosurfaces.

Category	Microdevices implications for highly curved bio-surfaces	
	DAYS-fluid (this work)	Sugar-transfer
Trigger stimulus	**Mechanical**<br>(stress-responsive,<br>ultra-low yield stress, ~tens of Pa)	**Thermal**<br>(melting at ≥80 °C)
	**Good**<br>Safe, non-invasive transfer on fragile biosurfaces (e.g., skin, egg yolk).	**Bad**<br>Local heating or burns on soft tissues.
Viscosity in operating state	**Low** and tunable under applied stress	**High** and temperature-dependent
	**Good**<br>Minimization of interfacial stress on microdevices	**Bad**<br>Transmission of stress and distorts fragile electronics

Supplementary Table 4 | Four fundamental technical distinctions between DAYS-fluid and sugar-transfer. Schematic comparison of DAYS-fluid and sugar-transfer illustrating differences in activation stimulus, microstructural transition, rheological behavior, and detachment mechanism[21-25].

Aspect	DAYS-fluid (this work)	Sugar-transfer
**(i) Activation Trigger**	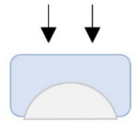 **Stress-responsive** Low mechanical stress	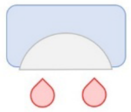 **Thermally responsive** Heating and cooling cycles
**(ii) Transition pathway**	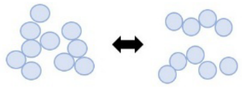 **Microstructure level** Rearrangement within a silica–water network	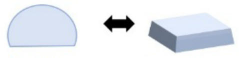 **Molecular level** Phase transition similar to ice · water transformation
**(iii) Rheological behavior in the fluidic state**	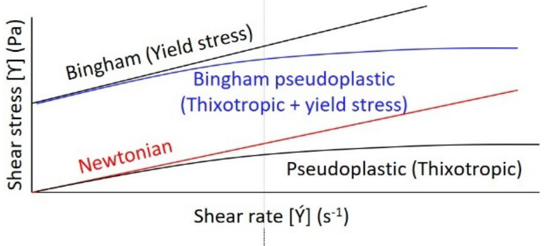 **Non-Newtonian** Viscosity and yield behavior affected by applied stress	**Newtonian** Viscosity constant and independent of applied stress
**(iv) Removal mechanism**	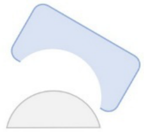 **Surface-localized** adhesion switching	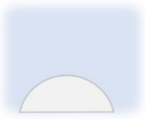 **Bulk dissolution** in water

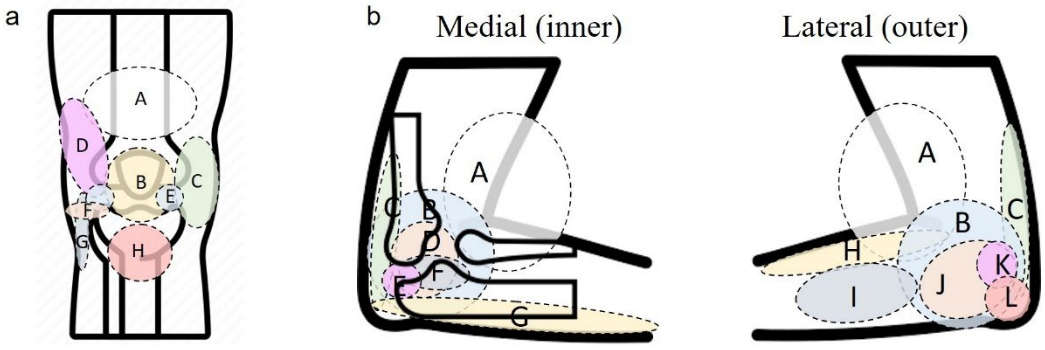

Supplementary Fig. 1 | Pain distribution map of convex joint. a, Pain diagram of knee joint**.** A Quadriceps tendonitis, B Patellofemoral pain syndrome, C MCL sprain arthritis, D ITB syndrome, E Osteochondritis dissecans, F Lateral Meniscus tear, G LCL injury, and H Patellar tendonitis. **b,** Pain diagram of elbow. A Biceps tendonitis, B Hyperextended elbow, C Triceps tendonitis, D MCL sprain, E elbow apophysitis, F Golfer's elbow, G Cubital tunnel syndrome, H Brachioradialis pain, I Radial tunnel syndrome, J Tennis elbow, K Radiohumeral bursitis, and L Olecranon bursitis [26-27].

Supplementary Note 2 | Mechanism of rheological behavior in DAYS-fluid

The rheological behavior of the DAYS-fluid originates from the dynamic hydrogen-bond network between fumed silica nanoparticles and surrounding water molecules (Supplementary Fig. 2).

In the solid-like state, silanol (Si–OH) groups on the silica surface form dense, multivalent hydrogen-bond interactions with neighboring water molecules. These interactions generate a space-spanning, percolated microstructure that immobilizes the nanoparticles and endows the material with yield-stress–driven elasticity. In this state, the fluid behaves mechanically like a soft solid: it resists deformation under low stress, maintains its shape over time, and provides structural integrity capable of supporting lightweight electronic devices without flow or sagging. This behavior is analogous to jammed colloidal or gel-like materials in which particles are physically locked within a hydrogen-bonded network[29-31].

In the liquid-like state, when external shear or compressive stress exceeds the yield stress, the hydrogen-bond network is temporarily disrupted. The nanoparticles become unlocked from the jammed configuration and are free to reorganize along the direction of applied stress, enabling viscous flow. Under this condition, the material transitions into a deformable, shear-thinning fluid that can spread, adapt to underlying topographies, and conformally flow around curved or undercut geometries. Once the applied stress is removed, rapid re-association of water molecules with silanol groups restores the hydrogen-bond network, returning the system to its solid-like, load-bearing configuration without structural degradation[29-31].

This reversible stress-induced transition differs fundamentally from the conventional solid–liquid phase transition observed in materials such as water, where melting involves a thermodynamic change in molecular order. Instead, yield-stress fluids such as DAYS-fluid undergo a **mechanical** transition controlled by network rearrangement rather than thermal energy. This behavior is similar to widely known thixotropic systems, including toothpaste, paint, or cosmetic gels, which flow under shear but recover a solid-like structure at rest.

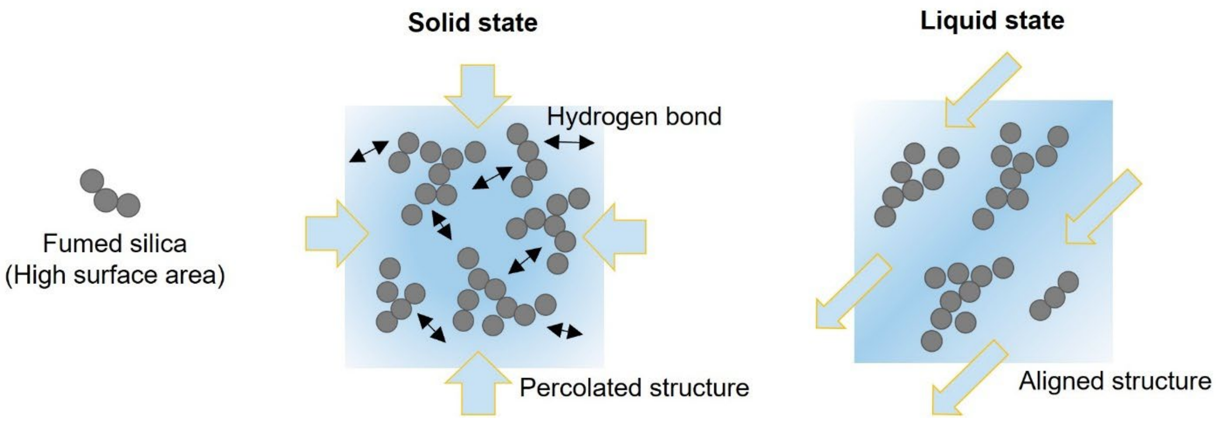

Supplementary Fig. 2 | Mechanistic schematic of the reversible phase transition in DAYS-fluid. The reversible solid–liquid like transition of DAYS-fluid is governed by the dynamic hydrogen-bond network formed between fumed silica nanoparticles and water molecules. *Resting state:* In the absence of external stress, a percolated hydrogen-bonded network between silanol (Si–OH) groups and water molecules forms a jammed, solid-like microstructure exhibiting yield-stress behavior. *Shear-induced flow state:* When external stress exceeds the yield stress, the hydrogen-bond network is temporarily disrupted, allowing the nanoparticles to flow past one another and the fluid to behave in a viscous, deformable state.

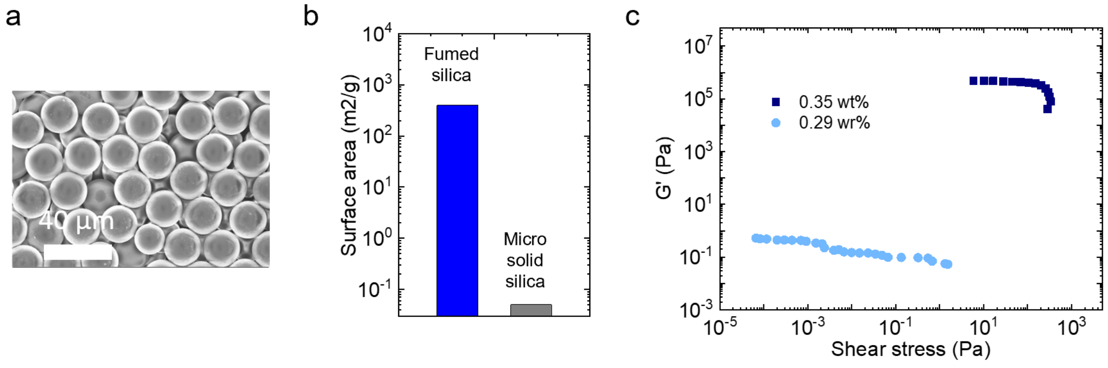

Supplementary Fig. 3 | Morphological and rheological characteristics of yield-stress fluids incorporating micro solid silica particles. a, Scanning electron microscopy (SEM) image of micro solid silica particles, showing uniform spherical morphology with a mean diameter of ~20 µm. Scale bar denote 40 µm. Surface area of 20 µm particles was calculated using diameter, while the value for fumed silica was taken from the manufacturer's specifications. **b,** Comparison of specific surface areas between fumed silica (used in DAYS-fluid) and micro solid silica particles, highlighting the ~$10^3\times$ higher surface area of fumed silica, which significantly contributes to yield-stress behavior through increased interparticle network formation[2]. **c,** Storage modulus (G') of silica-based fluids as a function of solid silica concentration, demonstrating that micro solid silica requires significantly higher loading to induce comparable elastic response, indicating poor network formation capability relative to fumed silica. These results support the use of fumed silica as a more effective rheological modifier in achieving the desired yield-stress behavior for stable and decoupled transfer. Source data are provided as a Source Data file.

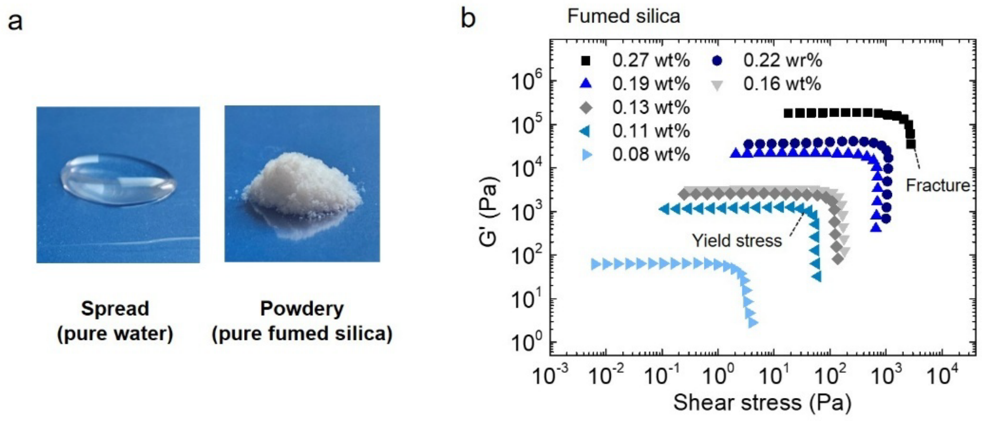

Supplementary Fig. 4 | Rheological behavior of aqueous suspensions containing fumed silica particles. a, Representative photographs of pure deionized water (left) and as-received fumed silica powder (right). **b,** Storage modulus (G′) as a function of fumed silica concentration, showing a sharp increase in elasticity with increasing particle content. The data demonstrate that even low concentrations (~0.1–0.2 wt%) are sufficient to induce a solid-like yield-stress behavior, due to the high surface area and strong interparticle interactions of fumed silica. This tunable rheological profile underpins the design of DAYS-fluid with desired flow-deformation decoupling characteristics. Source data are provided as a Source Data file.

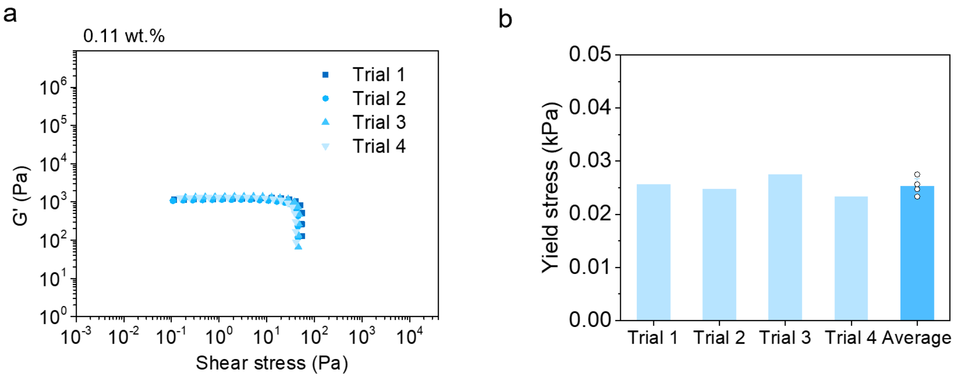

Supplementary Fig. 5 | Reproducibility of the yield stress measurements for DAYS-fluid. a. Storage modulus (G′) as a function of applied shear stress for 0.11 wt.% DAYS-fluid, measured from four independent samples. All samples exhibit consistent solid-like elasticity and a distinct yielding transition, where the storage modulus sharply decreases as the applied stress exceeds the yield point. **b,** Statistical summary of the yield stress values for the four samples, confirming high reproducibility across independent measurements (mean ± s.d. = 0.0253 ± 0.00135 kPa, n = 4 independent experiments). These results demonstrate the consistency and stability of the rheological behavior of the 0.11 wt.% DAYS-fluid under ambient conditions. Source data are provided as a Source Data file.

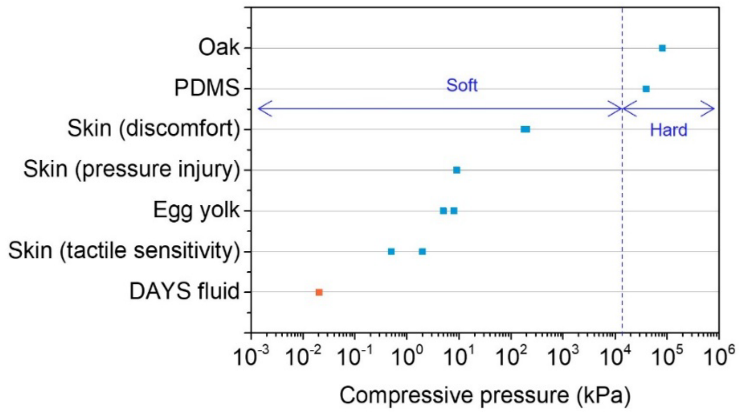

Supplementary Fig. 6 | Comparison of compressive pressure thresholds across biological and synthetic materials. Summary plot comparing representative compressive pressure levels for diverse biological and synthetic materials. Data points correspond to: the yield stress of DAYS-fluid, the skin tactile sensitivity threshold, the rupture pressure of egg yolk, the skin pressure threshold for pressure injury under ≥1 h exposure, the skin pressure discomfort threshold, the compressive failure pressure of PDMS (Sylgard 184, 10:1), and the perpendicular-to-grain compressive failure pressure of oak hardwood. These values illustrate the wide dynamic range of pressure tolerance across materials and highlight that the mechanical stress imparted by DAYS-fluid lies well below clinically relevant thresholds for sensitive biological tissues. [32-36]. Source data are provided as a Source Data file.

Supplementary Note 3 | Interfacial-level comparison of transfer-printing carriers on highly compliant and undercut substrates

To provide interfacial-level evidence of conformality and integrity, we performed a systematic comparative study using a highly compliant and undercut biological substrate: the mushroom cap shown in Fig. 1g-⑤. This geometry represents one of the most challenging cases for conformal electronic integration due to its low stiffness, negative curvature, and undercut morphology. Elastomer-based carriers, water-based hydrotransfer, high-viscosity liquid carriers (molten-sugar analogue), and the proposed DAYS-fluid were compared under identical transfer conditions (Supplementary Table 5). In addition to optical imaging, magnified confocal microscopy was used to directly visualize the device–substrate interface and assess local detachment and deformation.

Interfacial integrity was quantitatively evaluated using two complementary error metrics: detached area (%) and geometric strain (%). Detached area represents the fraction of the device that failed to adhere to the mushroom substrate. Geometric strain was estimated from deformation of serpentine interconnects by comparing magnified optical images with the original device geometry, following established geometry–strain correlations (Supplementary Fig. 7).

Elastomer-based carriers (stamps, pads, stretchable substrates): Elastomer-based transfer carriers require full-area, uniform contact to achieve stable adhesion. However, on highly compliant and undercut substrates such as mushroom tissue, the applied pressure primarily compresses the substrate rather than enabling conformal contact. Visualization using an ultrathin PDMS film confirms the absence of full-area contact, while increased pressure leads to mechanical damage and fracture of the substrate. These limitations are inherent to elastomer-supported stretchable carriers due to unavoidable backing-layer constraints.

Water-based carriers (hydrotransfer printing): Conventional hydro-dipping suppresses pattern spreading using a carrier film, but this configuration is incompatible with highly curved and undercut substrates that require substrate-free electronics. Without a carrier film, uncontrolled lateral water flow induces pattern spreading. During insertion onto curved substrates, fluid motion along the curvature generates strain, misalignment, and loss of pattern fidelity, as clearly observed in optical and confocal images.

High-viscosity liquid-based carriers (molten-sugar analogue): As illustrated in Fig. 2, high-viscosity liquid-based carriers rely on contact through a viscous liquid phase. Because direct

molten sugar transfer is impractical, a silica-particle-based high-viscosity fluid was used as an analogue. Interfacial imaging shows pronounced stretching of the electronics on the mushroom substrate, arising from deformation of the viscous fluid that transmits shear and compressive stresses to the device.

DAYS-fluid (this work).

In contrast, DAYS-fluid enabled uniform, full-area conformal contact across the entire mushroom surface, including undercut regions. Owing to its viscosity-controlled, stress-responsive flow, DAYS-fluid adapted locally to complex topographies without compressing the substrate or transmitting shear stress to the device.

Supplementary Table 5 | Comparative feasibility and interfacial integrity of transfer-printing carriers on highly compliant, undercut substrates. Photographic and confocal microscopy–based evaluation of representative transfer-printing carriers, including elastomer-based carriers (stamps, pads, stretchable substrates), water-based carriers, high-viscosity liquid carriers (molten-sugar analogue), and the DAYS-fluid developed in this work.

	Elastomer (ex. Stamp, Pad, Stretchable substrate)	Water	High viscosity liquid (ex molten sugar)	This work
**Optical image**	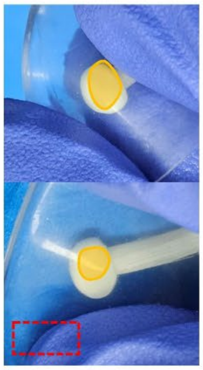<br>**Not Feasible**	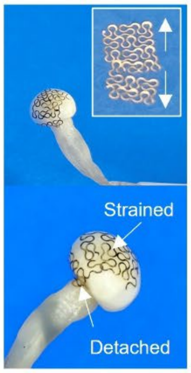<br>**Not feasible**	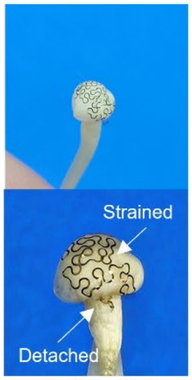<br>**Not feasible**	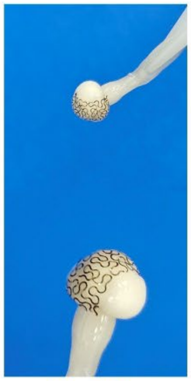<br>**Feasible**
**Confocal Microscopy**	**Not Feasible**	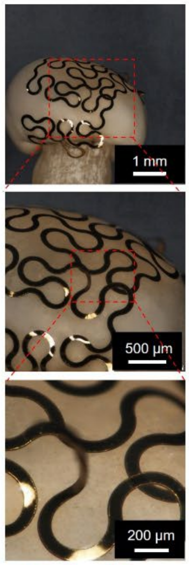<br>**Not feasible**	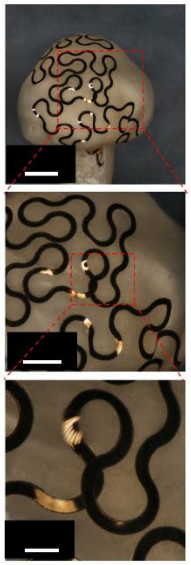<br>**Not feasible**	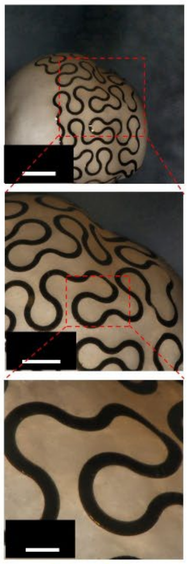<br>**Feasible**
**Mechanism**	- Require full-area contact for adhesion<br>- Substrate compression causes non-uniform contact or damage	- Uncontrolled fluid flow during floating or dipping	- Viscous flow transmits shear and compressive stress	- Detailed rheology control enables conformal contact
**Quantified interfacial error** – **Detached area (%)**	-	4.75%	8.14%	0%
**Quantified interfacial error** – **Geometric Strain (%)**	-	2.08%	5.27%	0%

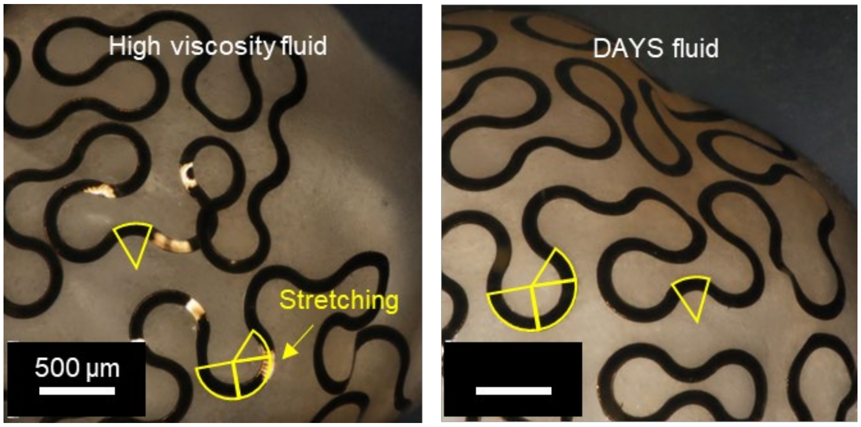

Supplementary Fig. 7 | Quantification of geometric strain as an interfacial error metric. Confocal microscopy images showing representative electrode patterns transferred using a high-viscosity fluid (left) and DAYS-fluid (right). Geometric strain was quantified by comparing the local curvature and arc geometry of the transferred serpentine traces with the original device design. For each curved segment, the apparent arc angle and radius were extracted from images and referenced to the undeformed geometry, enabling estimation of transfer-induced geometric strain (yellow guides). Scale bars denote 500µm.

Supplementary Note 4 | Substrate effect for adhesion mechanism of DAYS-fluid

DAYS-fluid adheres to substrates not through wetting or surface-energy–dependent interactions but through yield-stress–mediated mechanical gripping. Despite the ~3× variation in surface energy among polydimethylsiloxane (PDMS, ~20–24 mJ m^{-2}), polyimide (Kapton, PI, ~40–47 mJ m^{-2}), polyurethan (PU, ~35–45 mJ m^{-2}), styrene–ethylene–butylene–styrene (block copolymer) (SEBS, ~22–25 mJ m^{-2}), and glass (~65–75 mJ m^{-2}), the measured pull-off strength remained nearly constant (Fig. 3c), indicating that adhesion does not correlate with substrate surface energy or wetting behavior.

Instead, adhesion arises from the percolated fumed-silica network, which plastically deforms under gentle pressure and interlocks with nanoscale surface asperities, generating mechanically supported contact points determined by the fluid yield stress. This behavior aligns with the known adhesion characteristics of yield-stress fluids, where interparticle networks dominate interface mechanics rather than surface chemistry or wetting [30].

Upon water exposure, interfacial hydration locally disrupts hydrogen-bonded silica clusters, causing the mechanically supported interlocks to collapse and rapidly reducing adhesion to near zero. This switching process occurs regardless of substrate surface energy because adhesion is mechanically, not chemically, governed. These results confirm that DAYS-fluid operates through yield-stress-based gripping, enabling gentle, residue-free adhesion switching suitable for diverse biological and synthetic substrates.

Supplementary Note 5 | Environmental effects on adhesion-switching behavior of DAYS-fluid

To examine the influence of environmental parameters on the adhesion-switching dynamics of DAYS-fluid, we performed a series of controlled tests varying both relative humidity (RH) and temperature conditions. The experiments were conducted using a temperature–humidity-controlled chamber.

DAYS-fluid droplets (~100 μL) were first placed on a clean glass substrate and stabilized for 10 min under the target environmental condition. Following this preconditioning, water was gently applied to the top of each droplet and to trigger adhesion switching. The mobility of the droplet was then evaluated by tilting the substrate to 60° and applying three mechanical taps, identical to the protocol described in the main text (Fig. 3d–e). The moving head weight, defined as the mass of the detached portion of the droplet, was measured over time to quantify the detachment progression.

Humidity dependence (Supplementary Fig. 3a): At high RH% (≥60 RH%), water molecules readily diffuse into the interfacial region between the fluid and the substrate, rapidly disrupting the interfacial yield stress. This leads to fast adhesion loss and complete detachment within seconds after water exposure. Conversely, at low RH% (<4 RH0%), insufficient environmental water vapor slows hydration at the interface, delaying adhesion switching. The critical role of ambient water availability suggests that DAYS-fluid exhibits a humidity-accelerated interfacial hydration process.

Temperature dependence (Supplementary Fig. 3b): Temperature modulates adhesion-switching kinetics by altering the diffusion rate and molecular mobility of interfacial water. Under low-temperature conditions (3–5 °C, on an ice surface), the hydrogen-bond network within the fluid remains stable, resulting in sluggish detachment (~3 min). At elevated temperatures (80 °C, hot plate), rapid water diffusion and hydrogen-bond rupture cause almost instantaneous detachment (~5 s).

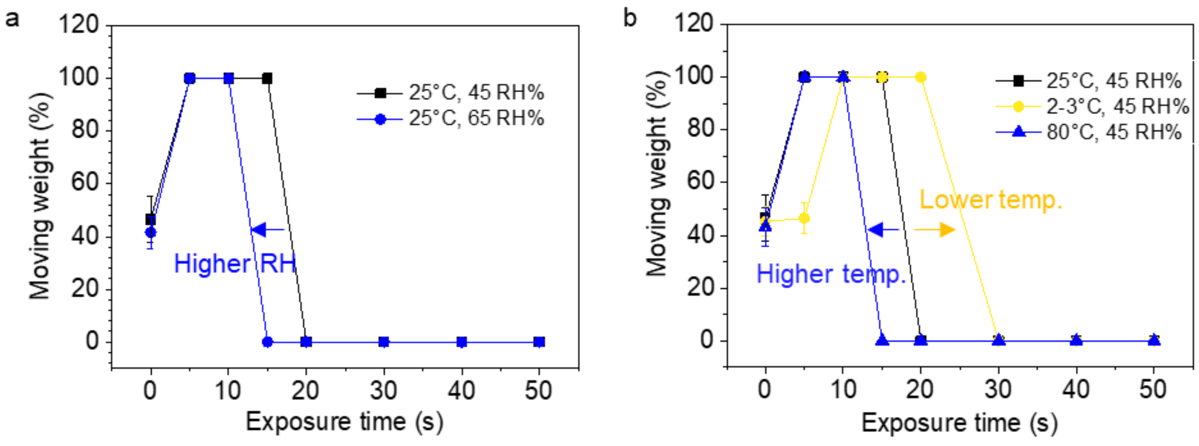

Supplementary Fig. 8 | Effect of environmental humidity and temperature on the adhesion switching kinetics of DAYS-fluid. a, Detachment time of DAYS-fluid under different relative humidity levels. Higher RH% accelerates adhesion switching by promoting interfacial hydration. **b,** Temperature-dependent detachment behavior. At 80 °C (blue), rapid water diffusion and disruption of hydrogen-bond networks enable complete detachment within ~5 s, whereas at 3–5 °C (yellow) reduced molecular mobility delays detachment to ~3 min. Error bars represent standard deviations from three independent measurements (mean ± s.d., n = 4 independent experiments). Source data are provided as a Source Data file.

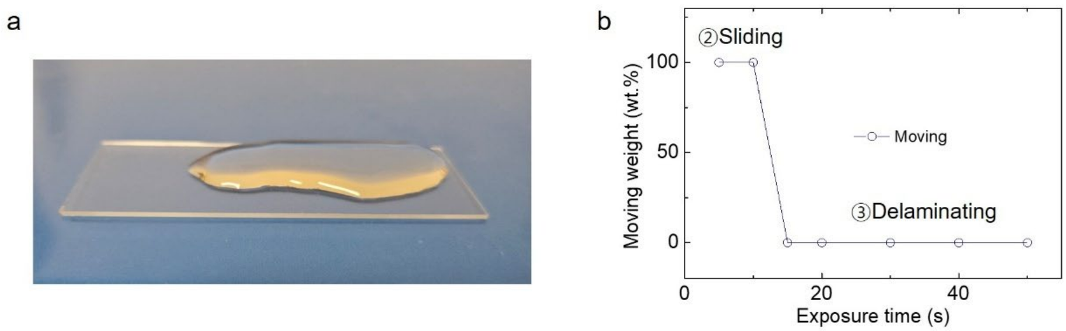

Supplementary Fig. 9 | Water-assisted mobility analysis of yield-stress fluid on wet slide glass. **a,** Photograph of a wet slide glass, with water uniformly applied to the surface to create a hydrated interface. This setup was used to investigate how interfacial water influences the detachment behavior of the yield-stress fluid. **b,** Graph showing the weight percentage of the moving portion of the fluid as a function of water exposure time (mean ± s.d., n = 4 independent experiments). Sliding occurs immediately followed by near-complete detachment at longer exposure times (>15 s), indicating rapid interfacial weakening by water penetration. Source data are provided as a Source Data file.

Supplementary Note 6 | Effect of internal water content on the adhesion-switching behavior of DAYS-fluid

To investigate the influence of intrinsic water content on the adhesion-switching performance of DAYS-fluid, a series of formulations with different internal hydration levels were systematically analyzed. Although the adhesion switching of DAYS-fluid is primarily triggered by external water diffusion at the fluid–substrate interface, the internal water content critically determines how this interfacial process progresses and whether the switching remains reversible and localized (Supplementary Fig. 10)

1. Overhydrated regime (particle content 0.08 wt.%)

When the water content of the fluid was excessively high, the DAYS-fluid exhibited a spreading morphology similar to that of pure water upon contact with the substrate (Fig. 1a, Supplementary Fig. 3b). In this overhydrated state, free water molecules dominate the interparticle interactions, weakening the hydrogen-bonded fumed silica network and reducing the yield stress. As a result, the fluid spreads uncontrollably across the surface and loses its cohesive integrity.

Under dry conditions, wetting at the fluid–substrate interface caused residual traces of fluid to remain on the surface. Under wet conditions, externally introduced water rapidly penetrated the bulk of the fluid, leading to complete dispersion and dissolution into the surrounding medium. This indicates that excessive internal water facilitates bulk disintegration rather than interfacial detachment, thereby undermining the controlled, reversible adhesion-switching process central to the DAYS-fluid mechanism.

2. Underhydrated regime (particle content 0.27wt.%)

When the internal water content was too low, the fluid exhibited a dry, dough-like morphology, as also shown in Fig. 1a and Supplementary Fig. 3b. Under these conditions, the interparticle hydrogen-bond network becomes overly rigid, preventing proper deformation and wetting. As previously discussed in *Mechanistic basis of DAYS-fluid for adaptive contact behavior*, such underhydrated formulations tend to crumble upon contact with the substrate, creating cracks and discontinuities at the interface. Consequently, adhesion remains weak and non-uniform, as the fluid cannot achieve sufficient conformal contact or mechanical interlocking. Even after exposure to water, these stiff, dough-like fluids maintain their structural rigidity.

3. Optimal hydration regime (particle content 0.11wt.%)

Between these two extremes lies an optimal hydration regime, in which the internal water content balances fluidity and structural cohesion. In this state, the hydrogen-bonded silica network remains continuous yet deformable, enabling controlled interfacial water diffusion and reversible adhesion switching. Water molecules introduced at the interface act as localized lubricants, promoting smooth, residue-free detachment without bulk deformation or dispersion.

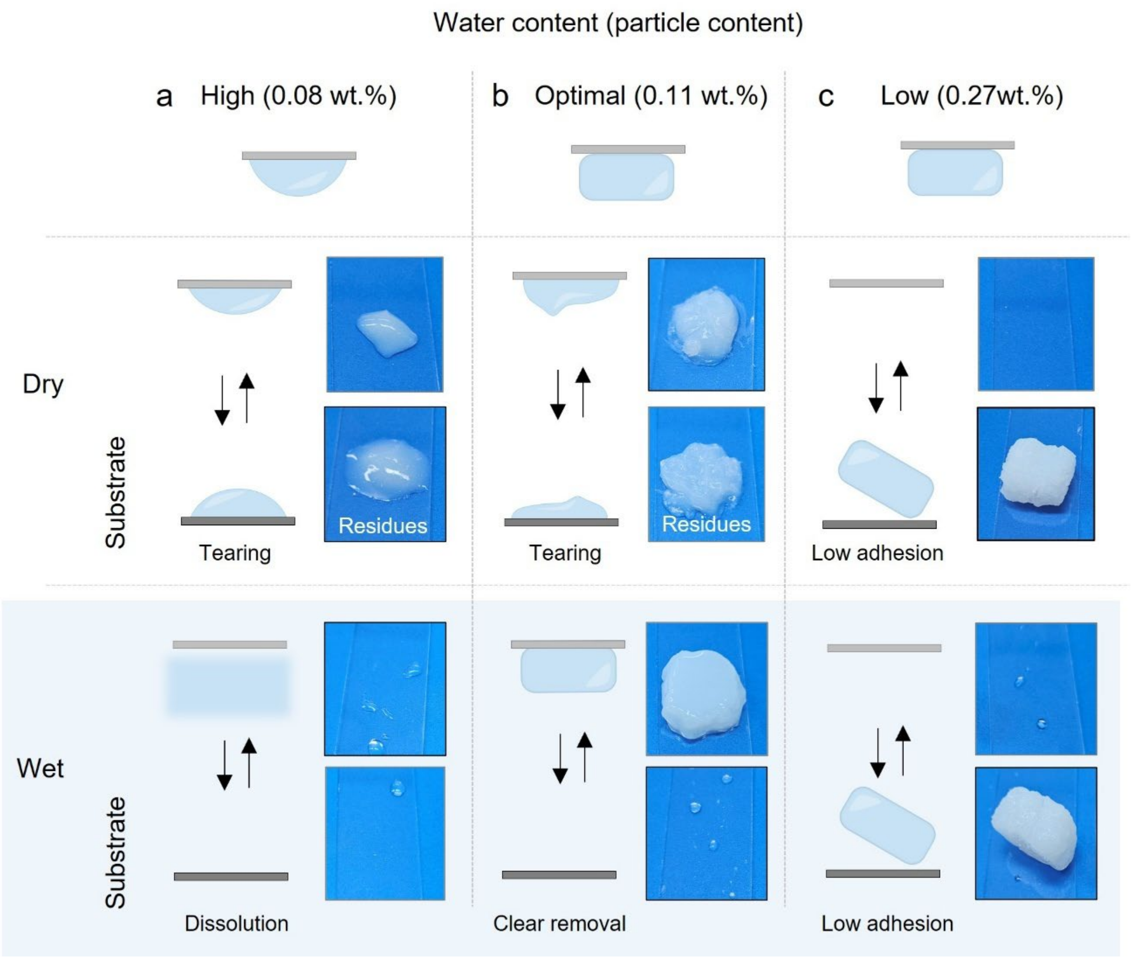

Supplementary Fig. 10 | Water-assisted adhesion switching behavior of the DAYS-fluid with different intrinsic water contents. Sequential schematic illustrations and optical images showing the adhesion and detachment behavior of the DAYS-fluid before and after water exposure: High water content (a), Optimal water content (b), and Low water content (c)

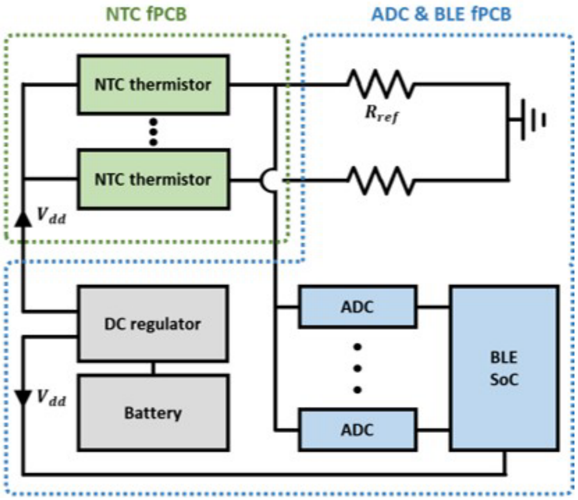

Supplementary Fig. 11 | Schematic circuit diagram of the thermal sensor array system. The system consists of two main flexible printed circuit boards (fPCBs): one for sensing (NTC fPCB) and the other for data acquisition and transmission (ADC & BLE fPCB). The NTC fPCB contains two negative temperature coefficient (NTC) thermistors that measure local skin temperature. These thermistors are connected to analog-to-digital converters (ADCs) located on the ADC & BLE fPCB, which digitize the temperature signals. A DC regulator and battery provide power to the system, while the digitized data is processed and wirelessly transmitted via a Bluetooth Low Energy system-on-chip (BLE SoC). This modular design allows for compact integration and real-time wireless temperature monitoring on soft or curved biological surfaces.

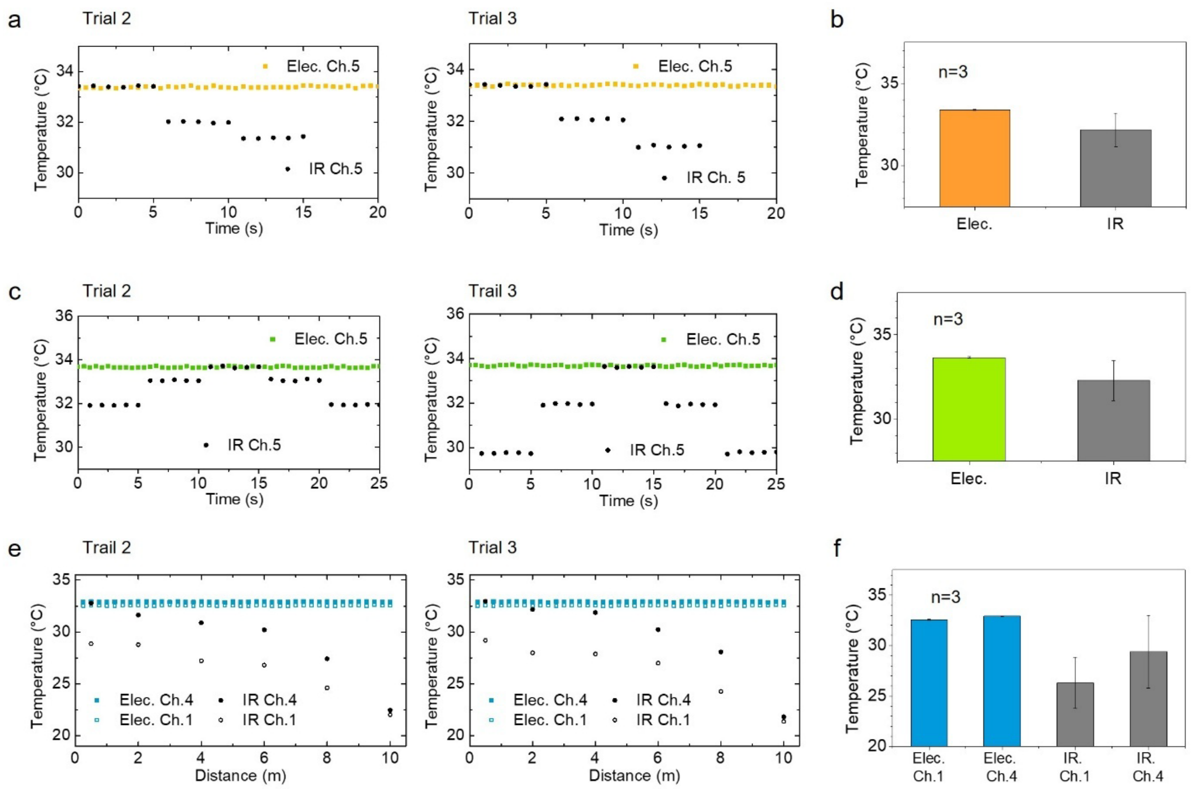

Supplementary Fig. 12 | Statistical analysis corresponding to Fig. 4e–g: real-time thermal sensing under various hand postures and camera distances. a, c, e, Real-time temperature measurements obtained from three independent cases (Trial 2 and 3) under varying hand postures (a, c) and different distances between the hand and IR camera (e). Each case represents an independent experimental condition showing consistent temperature profiles from DAYS-fluid–transferred sensors compared with IR imaging. **b, d, f,** Corresponding standard deviation plots of the temperature data (mean ± s.d., n = 3 independent experiments), demonstrating significantly smaller fluctuations and improved stability in the DAYS-fluid sensors relative to IR imaging. Source data are provided as a Source Data file.

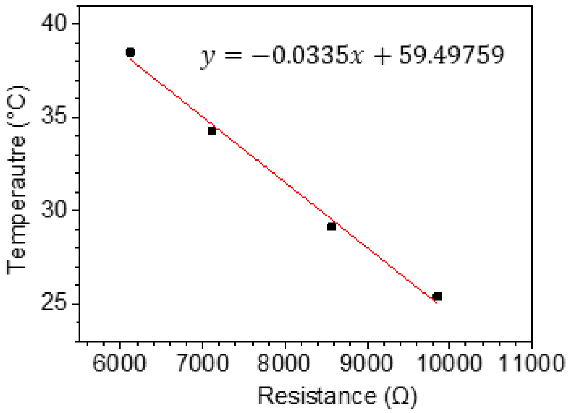

Supplementary Fig. 13 | Calibration curve of the thermal sensor array. Linear correlation between resistance (Ω) and temperature (°C) measured using the NTC thermistor-based sensor array. The calibration data were fitted with a linear regression (red line), showing high accuracy ($R^2 \approx 0.999$) across the physiological temperature range (25–45 °C), enabling precise skin temperature quantification during real-time monitoring. Source data are provided as a Source Data file.

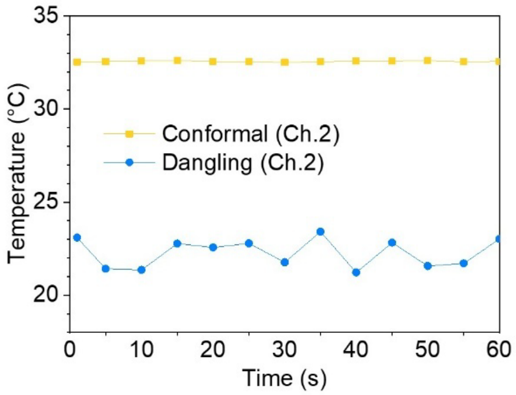

Supplementary Fig. 14 | Comparison of temperature stability during typing motion under conformal and non-conformal attachment. Real-time temperature measurements recorded from channel 2 of the finger-mounted thermal sensor during repetitive typing motion. The conformal condition (yellow), achieved using DAYS-fluid transfer, maintains a stable temperature profile with minimal fluctuation due to intimate contact with the skin. In contrast, the dangling condition (blue), produced by transfer with a high-viscosity fluid, exhibits pronounced fluctuations and transient deviations caused by partial detachment and motion-induced artifacts. Source data are provided as a Source Data file.

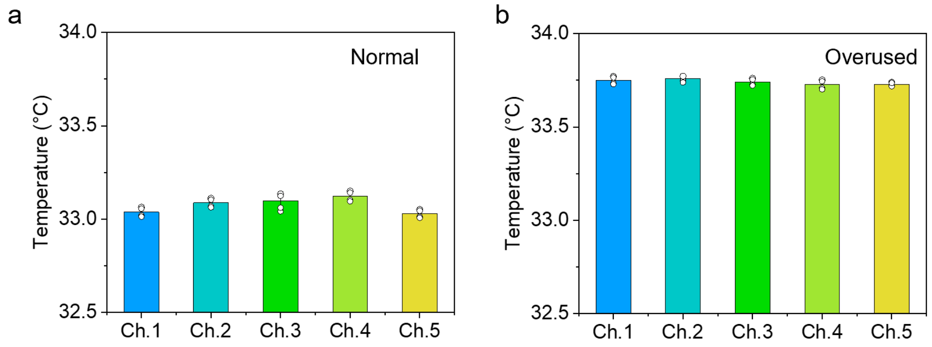

Supplementary Fig. 15 | Temperature comparison between normal (a) and overused (b) fingers measured by thermal sensor array. The bar graph shows the temperature values from each sensing channel (Ch.1 to Ch.5) placed on the left hand. To induce overuse, the subject repeatedly clenched and unclenched the hand for 5 min prior to measurement. All five channels exhibited a clear temperature elevation in the overused condition, with increases of approximately 0.6–0.85 °C compared to the normal condition (mean ± s.d., n = 4 independent experiments). Source data are provided as a Source Data file.

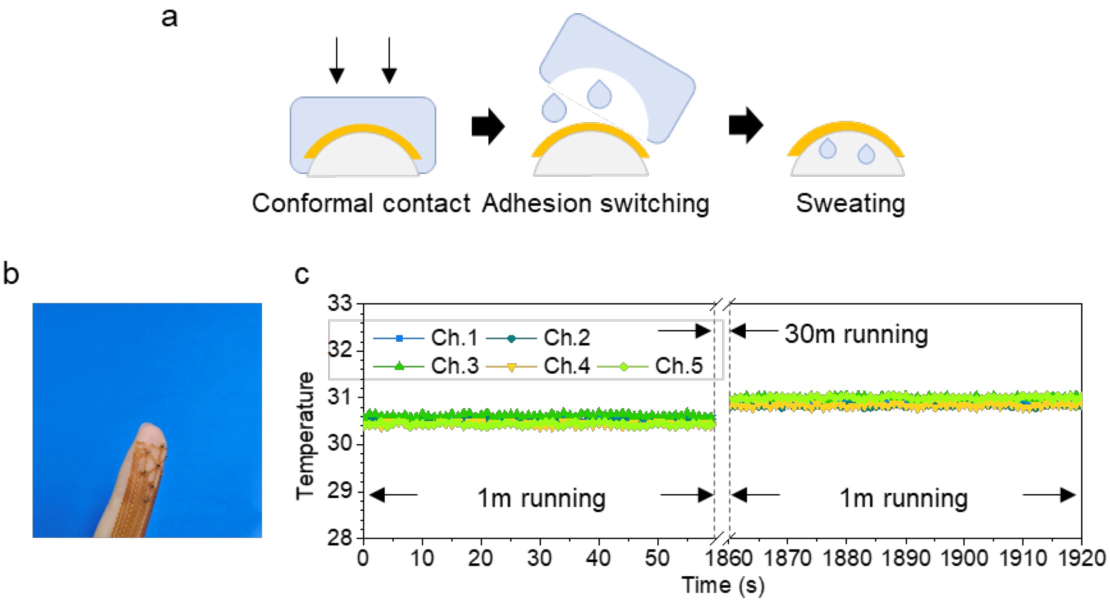

Supplementary Fig. 16 | Water-triggered adhesion-switching mechanism and real-time temperature monitoring using DAYS-fluid–transferred thermal sensors. a, Schematic illustration of the water-triggered adhesion-switching process of DAYS-fluid. **b,** Photograph of the real-time temperature sensing setup, where a thermal sensor array was conformally transferred to the underside of the index finger using DAYS-fluid. **c,** Real-time temperature monitoring recorded from channels (Ch. 1–Ch. 5) during sequential motion phases consisting of 1 min running, 30 min resting, and 1 min running. All channels maintain stable and continuous readings without baseline drift or signal spikes, confirming robust adhesion and reliable operation even under mild perspiration conditions. Source data are provided as a Source Data file.

Supplementary Note 7. Strain-sensor–based evaluation of transfer-induced mechanical and electrical integrity

To evaluate whether the DAYS-fluid transfer process preserves electrical performance for sensing modalities sensitive to interfacial strain and mechanical instability, we performed comparative transfer experiments using ultrathin resistive strain sensors on substrates with markedly different mechanical properties (Supplementary Fig. 17). Strain sensors were selected as a stringent benchmark because even minor transfer-induced deformation or interfacial instability immediately manifests as measurable electrical artifacts.

The strain sensor exhibits a linear and reproducible relationship between applied bending strain and relative resistance change ($\Delta R/R_0$), confirming its suitability for quantitative evaluation of transfer-induced mechanical effects (Supplementary Fig. 17). We first examined transfer behavior on a highly compliant and fragile biological substrate (lettuce leaf).

Consistent with the limitations of conventional transfer carriers discussed in Supplementary Table 5, existing transfer approaches induced significant mechanical and electrical degradation on the soft lettuce substrate (Supplementary Fig. 17c-g). All conventional transfer methods were performed following the same procedures described in Supplementary Table 5. Elastomer-based carriers failed to achieve conformal contact because applied pressure compressed and folded the substrate, resulting in incomplete adhesion and transfer failure (Supplementary Fig. 17c). High-viscosity liquid-based carriers, employed as an analogue of molten-sugar transfer, transmitted shear and compressive stresses during contact, leading to visible stretching of the strain sensor and pronounced resistance changes after transfer (Supplementary Fig. 17d and e). Water-based hydrotransfer caused uncontrolled fluid flow during floating and substrate dipping, producing pattern distortion, positioning difficulty, and severe electrical instability (Supplementary Fig. 17f and g).

In contrast, DAYS-fluid enabled gentle, deformation-decoupled transfer on the same soft lettuce substrate. The strain sensor transferred using DAYS-fluid exhibited negligible resistance change before and after transfer and maintained stable, reproducible strain-resolved signals during low-amplitude deformation (Supplementary Fig. 17h–j).

To further confirm generality across mechanical regimes, we evaluated strain sensing on a hard,

textured natural substrate (orange peel). Sensors transferred using DAYS-fluid showed unchanged resistance after transfer and stable, repeatable resistance modulation during repeated curvature changes (Supplementary Fig. 17k–m).

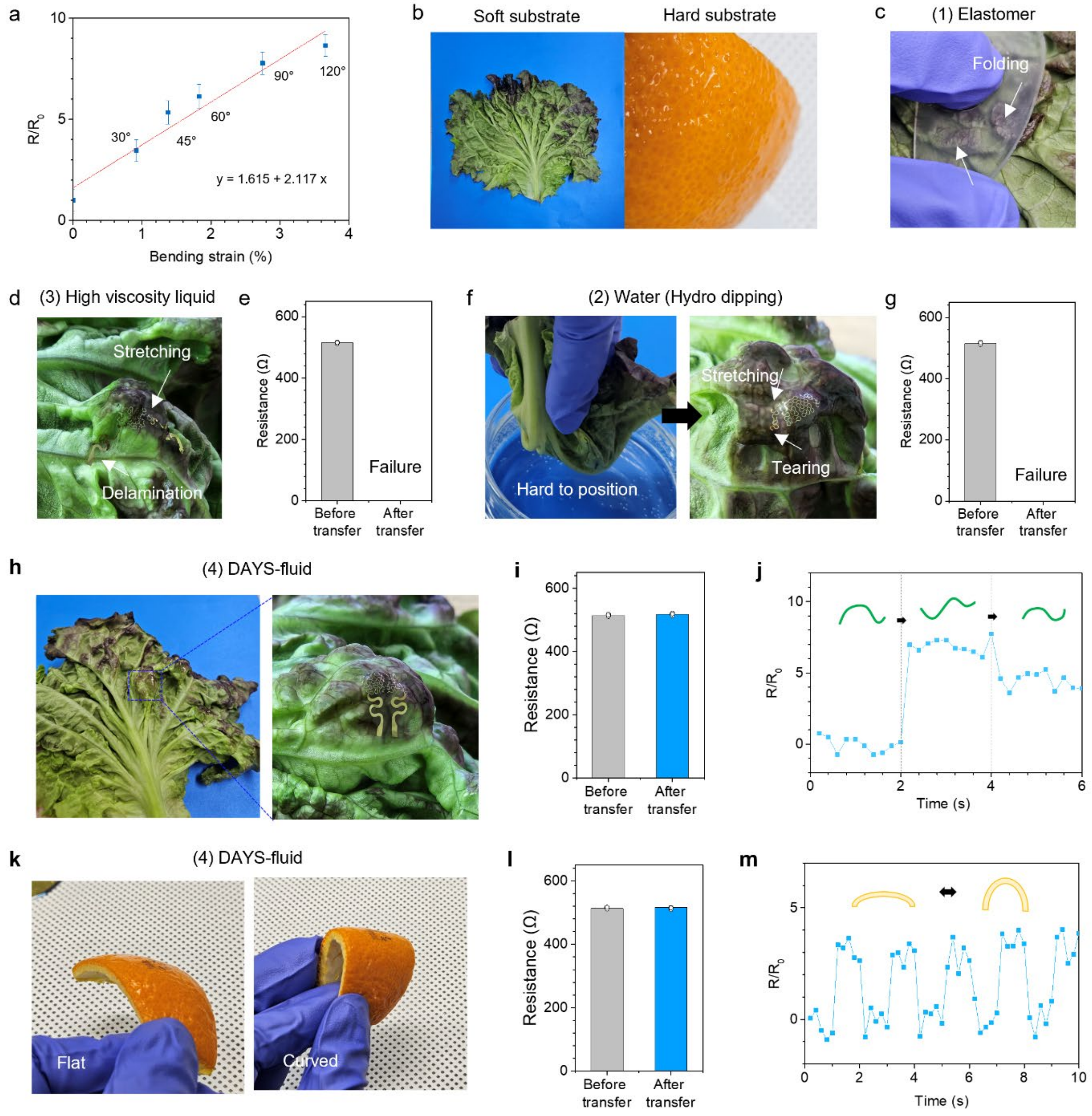

Supplementary Fig. 17 | Comparative evaluation of conventional transfer-printing carriers and DAYS-fluid for strain-sensor integration on soft and hard substrates. **a,** Calibration curve of the ultrathin strain sensor, showing a linear relationship between bending-induced strain and relative resistance change ($\Delta R/R_0$) (mean ± s.d., n = 10 independent experiments). **b,** Photographs

of representative soft substrates used for evaluation: lettuce leaf (soft, fragile). **c–g,** Transfer of strain sensors onto lettuce leaf using conventional transfer-printing carriers. **c,** Elastomer-based carriers (e.g., stamps, pads, stretchable substrates). During contact, the elastomer compresses and folds the soft lettuce substrate, preventing conformal contact and resulting in transfer failure. **d, f,** Photographs after transfer showing visible stretching of the strain sensor following transfer using a high-viscosity liquid-based carrier (d) and a water-based carrier (f). **e, g** Resistance of the strain sensor before and after transfer, indicating transfer-induced electrical instability for the high-viscosity liquid-based carrier (e) and the water-based carrier (mean ± s.d., n = 4 independent experiments) (g). **h–j,** Strain-sensing behavior on a *soft* biological substrate (lettuce leaf). Optical images of the sensor gently laminated onto the compliant lettuce surface (h). Resistance measured before and after transfer, confirming preserved device integrity (mean ± s.d., n = 4 independent experiments) (i). Dynamic resistance traces during low-amplitude deformation of the lettuce leaf, illustrating reliable sensing performance even on fragile, low-modulus substrates (j). **k-m,** Strain-sensing behavior on a *hard* natural substrate (orange peel). Optical images of the sensor conformally laminated onto the orange peel (k). Comparison of sensor resistance before and after DAYS-fluid transfer, showing negligible change (mean ± s.d., n = 4 independent experiments) (l). Time-resolved resistance signals during repeated curvature changes of the orange peel, demonstrating stable and reproducible strain detection on a stiff, highly curved surface (m). Source data are provided as a Source Data file.

Supplementary references

1. Purvis, A. *et al.* Photolithographic patterning of bihelical tracks onto conical substrates. *J. Micro/Nanolithogr. MEMS MOEMS* **6**, 043015 (2007).

2. Mohammed, M. G. & Kramer, R. All-printed flexible and stretchable electronics. *Adv. Mater.* **29**, 1604965 (2017).

3. Adams, J. J. *et al.* Conformal printing of electrically small antennas on three-dimensional surfaces. *Adv. Mater.* **23**, 1335–1340 (2011).

4. Carey, T. et al. Spray-coating thin films on three-dimensional surfaces for a semitransparent capacitive-touch device. *ACS Appl. Mater. Interfaces* **10**, 19948–19956 (2018).

5. Ko, H. C. *et al.* A hemispherical electronic eye camera based on compressible silicon optoelectronics. *Nature* **454**, 748–753 (2008).

6. Plovie, B. *et al.* Arbitrarily shaped 2.5D circuits using stretchable interconnects embedded in thermoplastic polymers. *Adv. Eng. Mater.* **19**, 1–8 (2017).

7. Kim, R. H. *et al.* Waterproof AlInGaP optoelectronics on stretchable substrates with applications in biomedicine and robotics. *Nat. Mater.* **9**, 929–937 (2010).

8. Yang, Y. *et al.* 3D multifunctional composites based on large-area stretchable circuits with thermoforming technology. *Adv. Electron. Mater.* **4**, 1–10 (2018).

9. Rich, S. I., Lee, S., Fukuda, K. & Someya, T. Developing the nondevelopable: creating curved-surface electronics from nonstretchable devices. *Adv. Mater.* **34**, 2106683 (2022).

10. Chen, X. *et al.* Wrap-like transfer printing for three-dimensional curvy electronics. *Sci. Adv.* **9**, eadi0357 (2023).

11. Lin, C. *et al.* Highly deformable origami paper photodetector arrays. *ACS Nano* **11**, 10230–10235 (2017).

12. Cheng, Q. *et al.* Folding paper-based lithium-ion batteries for higher areal energy densities. *Nano Lett.* **13**, 4969–4974 (2013).

13. Meitl, M. A. *et al.* Transfer printing by kinetic control of adhesion to an elastomeric stamp. *Nat. Mater.* **5**, 33–38 (2006).

14. Sim, K. *et al.* Three-dimensional curvy electronics created using conformal additive stamp printing. *Nat. Electron.* **2**, 471–479 (2019).

15. Wang, Y. *et al.* Electrically compensated, tattoo-like electrodes for epidermal electrophysiology at scale. *Nat. Biomed. Eng.* **7**, 501–510 (2023).

16. Yan, Z. *et al.* Thermal release transfer printing for stretchable conformal bioelectronics. *Adv. Sci.* **4**, 1700251 (2017).

17. Le Borgne, B. *et al.* Conformal electronics wrapped around daily life objects using an original method:

water transfer printing. *ACS Appl. Mater. Interfaces* **9**, 30345–30352 (2017).

18. Kim, D.-H. *et al.* Dissolvable films of silk fibroin for ultrathin conformal bio-integrated electronics. *Nat. Mater.* **9**, 511–517 (2010).
19. Giannakou, P., Tas, M. O., Le Borgne, B. & Shkunov, M. Water-transferred, inkjet-printed supercapacitors toward conformal and epidermal energy storage. *ACS Appl. Mater. Interfaces* **12**, 8456–8465 (2020).
20. Zabow, G. Reflow transfer for conformal three-dimensional microprinting. *Science* **378**, 648–653 (2022).
21. Yuk, H. et al. 3D printing of conducting polymers. *Nat. Commun.* **11**, 1604 (2020).
22. Zhao, S. et al. Additive manufacturing of silica aerogels. *Nature* **584**, 387–392 (2020).
23. Sugino, Y. & Kawaguchi, M. Fumed and Precipitated Hydrophilic Silica Suspension Gels in Mineral Oil: Stability and Rheological Properties. *Gels* **3**, 32 (2017).
24. Kim, H. et al. Embedded Direct Ink Writing 3D Printing of UV Curable Resin/Sepiolite Composites with Nano Orientation. *ACS Omega* **8**, 23554–23565 (2023).
25. Arif, Z. N. et al. Designing and transforming yield-stress fluids. *Curr. Opin. Solid State Mater. Sci.* **23**, 100758 (2019).
26. Shellie A. et al. Distinct patterns of variation in the distribution of knee pain, *Scientific Reports*, **8**, 16522 (2018)
27. Matre D. et al. Effects of localization and intensity of experimental muscle pain on ankle joint proprioception, *European Journal of Pain*, **6**, 4, 245-260 (2022)
28. Teasell, R. W. et al. Cardiovascular consequences of loss of supraspinal control of the sympathetic nervous system after spinal cord injury. *Arch. Phys. Med. Rehabil.* **81**, 506–516 (2000).
29. Yuk, H. et al. 3D printing of conducting polymers. *Nat. Commun.* **11**, 1604 (2020).
30. Barral, Q., et al. Adhesion of yield stress fluids. *Soft Matter* **6**, 1343–1351 (2010).
31. Johnson, K. O. & Phillips, J. R. *Tactile spatial resolution. I. Two-point discrimination, gap detection, grating resolution, and letter recognition. J. Neurophysiol***.** **46**, 1177–1191 (1981).
32. Stadelman, W. J., Newkirk, D. & Newby, L. *Egg Science and Technology* (4th ed., Taylor & Francis CRC Press, 2017). ISBN 978-0-203-75887-8.
33. Reswick, J. B. & Rogers, J. E. Experience at Rancho Los Amigos Hospital with devices and techniques to prevent pressure sores. In: Kenedi, R. M. & Cowden, J. M. (eds) *Bed Sore Biomechanics* (Strathclyde Bioengineering Seminars, Palgrave, London, 1976).

34. Nasir, S. H., Troynikov, O., Wong Lit Wan, D. & Zheng, Z. Assessing the pressure and thermal discomfort thresholds for designing of therapeutic gloves: a pilot study. *OBM Integr. Complement. Med.* **4**, 3 (2019).

35. Hao, Y., Xie, J., Xu, B., Hu, B., Zheng, Y. & Shen, Y. Tunnel elasticity enhancement effect of 3D submicron ceramic (Al_2O_3, TiO_2, ZrO_2) fibers on polydimethylsiloxane (PDMS*). J. Adv. Ceram.* **10**, 502–508 (2021).

36. Carmona Uzcategui, M. G., Seale, R. D. & França, F. J. N. Physical and mechanical properties of clear wood from red oak and white oak. *BioResources* **15**, 4960–4971 (2020).
