# Supplementary material for: Deformation- and damage-free transfer of soft electronics onto highly curved and fragile biological surfaces
Source: Nat Commun. 2026 Mar 26;17:4448. doi: 10.1038/s41467-026-70948-5 (PMC13183984; doi:10.1038/s41467-026-70948-5)
Supplement: Supplementary file 2 — Description of Additional Supplementary Information [file 41467_2026_70948_MOESM2_ESM.pdf]

Description of Additional Supplementary Files

File Name: Supplementary Movie 1
Description: Real-time demonstration of conformal contact between the ultrathin electronic device and a highly curved substrate using DAYS-fluid.

File Name: Supplementary Movie 2
Description: Real-time demonstration of removal of the DAYS-fluid via adhesion switching, leaving devices on the underlying substrate.
